# 3D Parametric and Nonparametric Description of Surface Topography in Manufacturing Processes

**DOI:** 10.3390/ma14081987

**Published:** 2021-04-15

**Authors:** Grzegorz Królczyk, Wojciech Kacalak, Michał Wieczorowski

**Affiliations:** 1 Faculty of Mechanical Engineering, Opole University of Technology, 45-758 Opole, Poland; 2 Faculty of Mechanical Engineering, Koszalin University of Technology, 75-900 Koszalin, Poland; wk5@tu.koszalin.pl; 3 Faculty of Mechanical Engineering and Management, Poznan University of Technology, 60-965 Poznan, Poland; michal.wieczorowski@put.poznan.pl

Surface topography has a profound influence on the function of a surface. In industrial practice, the geometric product specification is an important issue in multiple applications [1,2]. The measurement and characterization of the geometric features of machined parts are important when trying to determine the functional properties of surfaces and also in the control of process parameters during manufacturing [3]. However, there are many other areas of science or engineering where surface topography is critical to function. The emerging aim in a novel science approach is to determine the functional parameters of the generated surfaces. A well known fact is that surface topography can be described by the 3D parameters of a representative area of the selected surface and it is a result of the interaction between tool and surface [4] or tribological interaction [5]. Functional parameters can give us information about the future length of product life [6]. Proper parameter analysis can give us an answer to the question which parameter should control the entire manufacturing process [7]. Surface topography measuring instruments have advanced options which push measurement technologies to their limits. Therefore, deeper insight and a more comprehensive understanding of the performance of surface topography measurement solutions is needed [8]. Parametric description of surface is applied in various fields of science and can give as information about fundamental and practical approach. Królczyk [9] presents the *Ssk-Sku* map(Skewness-Kurtosis map) to better understanding the mixing process. The mixing process of particulate materials is a random process. The paper presents that in micro scale of the whole process many dependencies and behavioural conditions of the grains with respect to each other are observed. Gogolin et al. [10] presents surface topography as a method of pipe systems inspections. The authors present differences in geometry and a flow simulation to show flow nature and make validation of process by the PIV method. Pluta et al. [11] used surface topography in the area of biocompatibility assessment of polymer-ceramic connective tissue replacements; 3D samples topography has been given a broader picture of the surface of the analysed composite biomaterials.

Among the many methods of surface topography assessment [12], parametric and nonparametric methods can be distinguished. Parametric methods include the description of the surface topography using individual parameters from the S (Height Parameters) group. The most frequently described parameters in the open literature are: average roughness (*Sa*), root mean square roughness (*Sq*), maximum peak height (*Sp*), maximum valley depth (*Sv*), maximum height of surface (*Sz*), kurtosis (*Sku*) and skewness (Ssk). These parameters give a certain image of the surface, while a complementary image of the surface is obtained by presenting grouped parameters such as the set *Sp*, *Sv* and *Sz* or *Ssk*-*Sku* maps with the *Sq* parameter. Kacalak et al. [13] presents a new methodology for assessing the state of the surface in grinding using new sets of parameters. The aim of the work was to characterize their machining potential. The nonparametric methods of description of surface topography are surface morphology assessing, analysis of direction of surface structure, Abbott–Firestone curve (AFC), power spectral density (PSD) and fractal analysis. Generally, important applications of parametric analysis are an evaluation of the surface of technological machine parts for different manufacturing processes. There are also parameters whose values for each or most surfaces are significantly different, which means that they have a high ability to distinguish surfaces in relation to specific topography features [14]. In addition, sets of roughness parameters that differentiate a given set of surfaces depending on the criterion used. It is, therefore, important to determine both the individual criteria of the ability to differentiate surface features as well as to determine their combined impact on the selection of parameters with high classification capacity.

Overall look at surface topography in manufacturing processes with its parametric and nonparametric description can be divided into three main areas: numerical analysis, measurement devices and applications. From that point of view, in the Special Issue, there are a number of paper discussing numerical aspects of assessment of surface topography. Pawlus et al. [15] showed predicted parameters of the sum surfaces. They analyzed the relationships among the parameters of two contacted surfaces on parameters of equivalent surface, for which ordinates are sums of ordinates of both surfaces. Surfaces of various types (one- and two-process, isotropic and anisotropic, random or periodic) were studied. During the parameters selection in the research presented in the article, the authors present that the two pairs of parameters—*Sp/Sz* and *Sq/Sa*—can better describe the shape of the probability ordinate distribution of the analysed surface topography than the *Ssk-Sku* map. Additionally, they found that the RMS height Sq and the RMS slope *Sdq* parameters predicted with very high accuracy. The authors of reference [16] developed a method of the one-process profile valley modelling based on the two-process profile. They show that the one-process random profile is characterized by the Gaussian ordinate distribution by the standard deviation of the profile height and the length of correlation, addressing the problem of estimation of the correlation length of this one-process profile. As it was shown, the correlation length of the base one-process profile can be obtained on the basis of the vertical truncation of the measured two-process profile. The proposed procedure was validated for two groups of surfaces. The average error of the correlation length estimation in the research was not higher than 7%, while the maximum error was not larger than 14%. What is very important, the presented method in future applications can be extended to the simulated texture of areal surface topography. In another paper, [17], the authors present the developed limiting conditions of the presence of bimodal ordinate distribution, discussing two-process surfaces with plateau and valley parts. They are created by superimpositions of two one-process textures of Gaussian probability height distributions. Based on that, it is expected that the resulting two-process surface would have bimodal height probability distribution, but typically, two-process textures have unimodal ordinate distribution. Generated stratified textures and measured two-process surfaces of cylinder liners were taken into consideration. It was proved that conditions depend on the material ratio at the plateau-to-valley transition (*Smq* parameter) and the ratio of heights of the plateau and valley surface parts (*Spq/Svq* parameters). The bimodal ratio increased when the *Svq/Spq* ratio increased. In addition, when the Smq parameter is not lower than 50%, unimodal amplitude distribution exists. The results are functionally important because of the high tribological significance of the material ratio curve.

These problems are closely related to anisotropy of surfaces, that can be effectively quantified using multiscale approach. An example of such research is presented in [18], where Bartkowiak et al. discussed surface anisotropy and multiscale analysis after milling. Topographies were studied on two milled steel surfaces, one convex with an evident large scale, cylindrical form anisotropy, the other nominally flat with smaller scale anisotropies; a μEDMed surface, an example of an isotropic surface; and an additively manufactured surface with pillar-like features. The paper presents two methods for multiscale quantification and visualization of anisotropy. One method shows anisotropy in horizontal coordinates, the second uses multiple bandpass filters. Curvature tensors contain the two principal curvatures, i.e., maximum and minimum curvatures, which are orthogonal and their directions, at each location. The authors analysed texture aspect ratios (Str) and texture directions (Std) parameters. Analysed multiscale methods show changes in anisotropy with the scale on surface measurements with markedly different anisotropies. Changes of anisotropy with scale categorically failed to be detected by traditional characterization methods, while multiscale methods proved to be very useful. Directions of principal curvatures superimposed on height maps also elucidate anisotropies at specific scales. Different scales show the multiscale nature of different sorts of anisotropies. Continuing multiscale approach to surface topography, Marteau et al. [19] present a study with the morphological signature of roping to link roughness results with five levels identified by a visual inspection. Roping or ridging is a visual defect affecting the surface of ferritic stainless steels, assessed using visual inspection of the surfaces. The researchers show multiscale analysis of surface roughness of the Str parameter and used the autocorrelation function for the quantification. The use of the isotropy with the Sq parameter led to a clear separation of the five levels of roping. To obtain a gradation description instead of a binary one, a methodology based on the use of the autocorrelation function was created, that consisted of a low-pass filtering, segmentation of the autocorrelation into four stabilized portions and computation of isotropy and the root mean square roughness Sq on the obtained quarters of function. Both methodologies can be used to quantitatively describe surface morphology of roping in order to improve our understanding of the roping phenomenon. In addition, Eseholi et al. [20] present multiscale topographical analysis based on morphological information. In the article, the researchers evaluated the effect of scale analysis, as well as the filtering process on the performances of an original compressed domain classifier in the field of material surface topographies classification. Each of the surface profiles has been multiscale analysed using a Gaussian Filter and decomposed into three multiscale filtered image types: Low-pass (LP), Band-pass (BP) and High-pass (HP) filtered versions, respectively. The images are lossless compressed using the High-effciency video coding (HEVC) standard. Compared to conventional roughness descriptors, the HEVC-MD descriptors increase surfaces discrimination from 65% to 81%. The results demonstrated that the robust compressed-domain topographies classifier is based on multiscale analysis methodologies.

Surface topography or surface meshing to be more precise, can be also used to create an efficient path planning algorithm. This is presented in publication written by Xiao et al. [21]. Generally, path planning algorithms for automated fiber placement are used to determine the directions of the fiber paths, while the quality of the fiber paths determines the efficiency and quality of the automated fiber placement process. The authors proposed a method of the datum direction vector via a guide-line update strategy to make the path planning algorithm applicable for complex surfaces. Sub-surface boundary splicing and surface topology reconstruction algorithm were proposed and both the computational complexity reduction and the efficiency improvement of the algorithm were analyzed. Additionally, an accuracy analysis on the proposed algorithm was performed, to investigate the relationship between the triangulation parameters and distance deviation, angle deviation and algorithm efficiency. The analysis indicated that choosing appropriate triangulation parameters is crucial for generation of the fiber path with high accuracy and efficiency.

The second area discussed in this Special Issue of Materials is related to surface measuring devices and conditions that needs to be maintained. Here, Kaplonek et al. [22] present non-contact measurement techniques as a support to X-ray-based methods. The authors presented wear of coins, as their wear affects utility values, qualifying them as a legal tender in a country. In this paper, they presented measured and analyzed surfaces with advanced high-accuracy optical profilometry methods. The obtained results confirm the validity of the applied high-accuracy measurement systems in such an application. Furthermore, the analysis can be a significant source of information regarding the condition of coins in the context of maintaining their functional properties. Discussing environmental conditions necessary to perform proper measurements, Grochalski et al. [23] present the effect of thermal phenomena on areal measurements of surface topography using stylus profilometry. The measurements were implemented under variable environmental conditions. The influence of internal heat sources from profilometer drives and their electronic components was analyzed. For this purpose, a thermal chamber was designed and built. On the obtained data, the authors proposed the time after which the correct topography measurement can be started. The value of elongation in individual axes of the profilometer is different and it very much depends on the construction of the device, type of drives used and their location. The largest impact on the imaging of the surface topography has the displacement of the probe in the direction of the *Z*-axis. This displacement directly translates into the obtained value of the height of the measured surface. The thermal and geometrical stabilization times should be precisely determined before beginning a 3D surface measurement. It was shown that performing thermal stabilization of the profilometer significantly reduced surface irregularity errors.

Now, concentration is owed to applications. The most common ones are related to machining. In this field, Aslantas et al. [24] present experiments and predict surface topography after micro-turning, as a micro-mechanical cutting method used to produce cylindrical parts with small diameter, where a second operation such as grinding may be difficult. The scientists describe empirical relations between technological cutting parameters and surface topography of titan alloy, using a multi-objective optimization. The research was developed using the RSM method, while the scanning electron microscope (SEM) analysis was done on the cutting tools to observe abrasion and crater wear mechanism. The overall results depict that the feed rate is the prominent factor that significantly affects the responses in micro-turning operation. Struzikiewicz and Sioma [25] present selected practical problems related to the surface quality after machining of AlSi10MG alloy powder made by laser sintering. Thanks to surface topography they found the occurrence of breaches on the machined surface, which negatively influence on the surface quality. The cause of breaches and deformations on the machined surface is probably the structure of the surface layer of the sintered aluminum and the method and conditions of combining material particles during the laser sintering process. Surface topography has been presented based on 3D microscopic analysis. The results of the research on the effect of cutting parameters on the values of parameters describing the surface quality are also presented. Taguchi’s method was used in the research methodology. It is likely that there are areas with weaker material particle joints that were produced by melting and subsequently by combining metal powder particles. In another paper, Bartkowiak et al. [26] studied the state of surface topographies after electric discharge machining. The measured topographies consist of overlapping microcraters. For this, the authors used conventional ISO parameters, nonparametric motifs and multiscale analysis with curvature tensor. Motif analysis uses watershed segmentation which allows extraction and geometrically characterization of each crater. Curvature tensor analysis focuses on the characterization of principal curvatures and their function and their evolution with scale. Surfaces have been measured by focus variation microscopy. Strong correlations between the height of the crater, diameter, area and curvature was observed. The scientists proved a stronger correlation between conventional areal parameter related and heights dispersion. The approach presented in paper allows for extraction of information directly relating to the shape and size of topographic features. The results show experimentally that the microgeometry of surfaces created by EDM (Electrical Discharge Machining) is strongly affected by the discharge energy. Sutowska et al. [27] show surface topography after the impact of curvature of a shape cut out in a brittle material. The curvature of a shape, resulting from the size of the radius of the cutting head trajectory, is one of the key requirements necessary for ensuring the required surface quality of materials shaped by the abrasive water jet process. The manufacturing process used to generate the surface has been an abrasive water jet (AWJ). The results of the experiments confirmed that the effect of the curvature of the cut shape is an important factor from the efficiency point of view. The parameter used in this research was the total height of surface irregularities given by the St amplitude parameter. The obtained results of the experimental studies confirmed that the effect of the curvature of the cut shape is important from the point of view of the efficiency of the glass-based brittle material-cutting process using AWJ. An interesting way of machining is described in [28], where Singh et al. present the potential of magneto-rheological fluid-assisted abrasive finishing for generating precise surface topography of titan alloy for orthopedic applications. The corrosion performance of the finished samples has also been analyzed through simulated body fluid (SBF) testing. The corrosion analysis of the finished samples specified that the resistance against corrosion is a direct function of the surface finish. It has been found that the selected input process parameters significantly influenced the observed MR and Ra values at 95% confidence level. The morphological nonparametric analysis presented that the rough sites on the surface have provided the nuclei for corrosion mechanics that ultimately resulted in the shredding of the appetite layer. Overall, the results highlight that the MRF-AF process is highly suitable for producing nano-scale finishing of the biomedical implants made of high-strength β-phase Ti-Nb-Ta-Zr alloy. Abrasion and wear are connected with all the types of machining. The effect of cryogenic treatment and post tempering on the behavior of abrasive wear, in the presence of angular quartz sand of rotavator blade is presented in [29]. Rotavator blades are prone to significant wear because of the abrasive nature of sand particles. In this research, the authors show that cryogenic treatment has caused an improvement in the abrasive wear resistance and microhardness compared to untreated material due to enhancement in hardness. Cryogenic treatment has caused an improvement in the abrasive wear resistance and microhardness, compared to untreated material due to enhancement in hardness, the conversion of retained austenite into martensite and the precipitation of secondary carbides in boron steel after exposure to cryogenic temperature. The additional cost was incurred due to cryogenic treatment, but economic analysis justifies the additional cost of that operation. Tool wear analysis was analyzed by Bazan et al. [30], who presented a comparison of surface microgeometry of the grinding wheel by stylus and optical profilometry. In the article, the authors propose a new methodology for determining the average level of binder, which allows the definition of the cut-off level required to separate from the measurement data. This methodology allows one to track changes in characteristic parameters computed from measurements of surface topography in the above-mentioned areas due to different wear processes. Among the parameters for assessing the geometric features of the surface, there are many that do not differentiate individual surfaces in the analyzed set or do not distinguish a significant group. Measurements of the active surface microgeometry of the grinding wheel are commonly used to obtain a cloud of points representing the surface of the examined tool. The research was based on the analysis of data obtained from measurements of single-layer grinding wheels using the replica technique. Discussing applications, finally, Krawiec et al. [31] propose thermography as a non-contact diagnostic tool for assessing drive reliability. The researchers present research during the operation of the belt transmission with a heat-welded thermoplastic polyurethane V-belt. The V-belt temperature changes depending on the braking torque load at different values of the rotational speed of the active pulley, which were adopted as diagnostic characteristics. In this article, the surface morphology of the polyurethane belts has been evaluated based on microscopic tests. The surface topography of the samples was determined by scanning electron microscopy (SEM) and optical profilometry. On the microscopic images, there was a lack of traces of cracks and scratches on the surface, for deeper analysis the authors used observations performed on an electron microscope. It was found that the most favorable operating conditions occurred when the temperature values of active and passive connectors were similar and the temperature difference between them was small.

Already, this short presentation shows how important surface topography is for various fields of science. It also presents many different approaches to the assessment of asperities. This makes the 3D analysis of surfaces in micro scale a very important topic, aiming to create better mechanisms, systems and solutions for our everyday life. The the Industry 4.0 strategy is discussed and different sensors monitoring the states of machines and communicating with other ones are considered. Artificial intelligence will govern many problems of today which are too complicated for operators. Here, filtration methods and procedures are a great example, bearing in mind a large and growing number of options that can be chosen for certain applications. Still, all the new ideas do not change the fact that, in order to obtain proper results, surface topography simply must be manufactured.

## Data Availability

Data sharing is not applicable to this article.
